# Supplementary material for: Analytical and clinical validation of an amplicon-based next generation sequencing assay for ultrasensitive detection of circulating tumor DNA
Source: PLoS One. 2022 Apr 29;17(4):e0267389. doi: 10.1371/journal.pone.0267389 (PMC9053827; doi:10.1371/journal.pone.0267389)
Supplement: S1 File — (PDF) [file pone.0267389.s017.pdf]

Minimal Data Set for Figures

	Mean	Upper 95% CI	Lower 95% CI	n samples
**Fig 2D**				
Ex19del	94.59	97.88	86.91	78
L858R	98.33	99.91	91.14	65
T790M	96.3	99.81	81.72	32
G719X/L861Q	100	100	68	11
**Fig 2E**				
Ex19del	98.58	99.45	96.4	285
L858R	97.87	99.09	95.12	296
T790M	98.48	99.35	96.48	329
G719X/L861Q	99.14	99.76	97.49	347

**Fig 5A**	**n with no detectable variants**	**n with ≥1 variant, all >0.3% VAF**	**n with ≥1 variant ≤0.3% VAF**
Lung	215	310	305
Breast	64	83	93
Colorectal	32	49	54
Pancreas	16	30	35
Gynecological	20	13	14
Unknown Primary	6	15	15
Bile Duct	3	17	14
Prostate	11	4	17
Liver	3	7	10
Gastroesophageal	4	8	5
Nasopharyngeal	5	5	4
Urothelial	1	5	7
Kidney	4	0	5
Others	4	1	3
Skin	6	0	1
Blood	2	2	3
Sarcoma	4	1	1
Head and Neck	4	0	1
CNS	1	2	2
Peritoneal	1	1	3
Thyroid	0	1	1
Thymus	0	1	1
Screening (including suspected cancers)	62	6	3

Fig 5D	n missense	n nonsense	n frameshift	n in-frame	n splice	n fusion	n deletion	n amplification
EGFR	309		1	227				23
TP53	236	34	45	11	36			
KRAS	63							2
PIK3CA	56			1				
BRCA2	23	2	11		1		3	
MET	11		2		7			11
SMAD4	23	6	2					
ERBB2	5			22				3
BRAF	25	1	1	2				
BRCA1	19	2	4	2	1			
KEAP1	15	1	4	2				
ALK	4		1			12		2
APC	7	6	3	2				
NF1	7	1	4	1	2			
PTEN	5	1	5	2	1		1	
RB1	3	6	3		1		2	
CTNNB1	12			1	1			
CDKN2A	3	5	1	1			3	
NRAS	13							
NFE2L2	8	1	1	1	1			
SF3B1	10			1				
RET			1	1		7		
GNAS	8							
ROS1	3					5		
JAK2	6							

Fig 5E	n no actionable targets	n potential actionability	n resistance mutations	n ≥1 druggable target
Top	104	39	26	446

**Fig 5E**	**n**
Bottom	
EGFR ex19del/L858R/L861Q/G719X/S786I	350
EGFR T790M	81
EGFR amp	23
MET amp/ex14sk	22
KRAS G12C	19
EGFR ex20ins	19
ALK fusions	12
BRAF V600E	10
RET fusions	6
ROS1 fusions	5

**Fig 5F**	**n**
**ALK inhibitor**	
Amp	2
G1202R	2
L1196M	1
**1G/2G EGFR TKI**	
EGFR T790M	67
**Osimertinib**	
EGFR C797S	23
PIK3CA alterations	16
EGFR amp	13
KRAS alterations	9
MET amp	7
EGFR L718/G796	6
BRAF G469/V600	5
CCND1 del/CDKN2A amp	3
CCDC6-RET fusion	3
FGFR3-TACC3 fusion	1
NF1 alterations	1

Fig 5G	n missense	n nonsense	n frameshift	n in-frame	n splice	n fusion	n deletion	n amplification
TP53	69	8	25	5	7		3	
PIK3CA	84		1	3				1
ESR1	65							2
BRCA2	10	1	5	2	3		5	
ERBB2	9							16
PTEN	4	4	7		1		1	
GATA3	2		9		4			1
RB1	2	7	1	1	2		3	
SMAD4	10		1	1	1		3	
BRCA1	11		3	1				
APC	10	1	2					
AKT1	9							
MYC		1						7
CCND1								7
GNAS	4							1
KRAS	5							
